# Mechanical Properties Optimization of Poly-Ether-Ether-Ketone via Fused Deposition Modeling

**DOI:** 10.3390/ma11020216

**Published:** 2018-01-30

**Authors:** Xiaohu Deng, Zhi Zeng, Bei Peng, Shuo Yan, Wenchao Ke

**Affiliations:** 1 National Local Joint Engineering Laboratory of Intelligent Manufacturing Oriented Automobile Die & Mold, Tianjin University of Technology and Education, Tianjin 300222, China; dengxh@tute.edu.cn; 2 Tianjin Key Laboratory of High Speed Cutting and Precision Machining, Tianjin University of Technology and Education, Tianjin 300222, China; 3 School of Mechatronics Engineering, University of Electronic Science and Technology of China, Chengdu 611731, China; bpe188@gmail.com (B.P.); 201722080301@std.uestc.edu.cn (S.Y.); 201721080101@std.uestc.edu.cn (W.K.); 4 Institute of Electronic and Information Engineering of UESTC in Guangdong, Dongguan 523808, China

**Keywords:** fused deposition modeling, polyether-ether-ketone, tensile properties, flexural properties, impact properties

## Abstract

Compared to the common selective laser sintering (SLS) manufacturing method, fused deposition modeling (FDM) seems to be an economical and efficient three-dimensional (3D) printing method for high temperature polymer materials in medical applications. In this work, a customized FDM system was developed for polyether-ether-ketone (PEEK) materials printing. The effects of printing speed, layer thickness, printing temperature and filling ratio on tensile properties were analyzed by the orthogonal test of four factors and three levels. Optimal tensile properties of the PEEK specimens were observed at a printing speed of 60 mm/s, layer thickness of 0.2 mm, temperature of 370 °C and filling ratio of 40%. Furthermore, the impact and bending tests were conducted under optimized conditions and the results demonstrated that the printed PEEK specimens have appropriate mechanical properties.

## 1. Introduction

Poly-ether-ether-ketone (PEEK) has received widespread attention due to the good mechanical properties, biocompatibility and elastic modulus closing to human bones, and is considered one of the most promising bone repair materials [1,2]. As demands of PEEK increase, additive manufacturing (AM) is starting to be used in the forming process of PEEK components.

In the past decades, selective laser sintering (SLS) is the most popular additive manufacturing technology for PEEK [3,4]. However, the high cost, poor penetrability and concentrated beam of laser restrict it from sintering large areas or laminates. So far, fused deposition modeling (FDM) is one of the most mature, popular and fastest growing three-dimensional (3D) printing methods, which are employed to produce large size medical implants. Valentan et al. [5] reported that FDM has been an alternative method to process PEEK parts.

The mechanical properties of FDM parts are mainly dependent on production parameters such as printing temperature, printing speed, scanning path, nozzle diameter, layer thickness, chamber temperature, and filling ratio [6]. Several studies have been carried out to evaluate various mechanical parameters of polylactic acid (PLA) and acrylonitrile butadiene styrene (ABS), including tensile, compressive, flexural and impact strength. Gordon et al. [7] presented the effects of several production parameters on the mechanical properties of FDM specimens with PLA. Luzanin et al. [8] discussed the influence of the layer thickness, deposition angle and infill on the maximum flexural force in FDM specimens made of PLA. Ismail et al. [9] developed the functional relationship between the FDM parameters (raster angles and part orientations) and strength, surface roughness and production cost of specimens. Pfeifer et al. [10] developed a theoretical relationship among four FDM printing parameters (extrusion rate, nozzle travel speed, layer height and path width). Chacon et al. [11] investigated the influences of build orientation, layer thickness and feed rate on the mechanical performance of PLA specimens.

Compared to the PLA and ABS wires, PEEK has a higher melting point, it is liable to cause excessive thermal stress (unevenly distributed between layers) and thermal cracks. Therefore, PEEK material needs specific parameter setting to adapt its own characteristics [12]. Wu et al. [13,14] investigated the mechanical strengths and the thermal deformation performance of PEEK material with different printing parameter settings in FDM. PEEK can be processed into a filament, and specimens can be manufactured by using several build orientations and extrusion paths. Kazi et al. [15] investigated the relationship between various thermal processing conditions (the ambient temperature, the nozzle temperature and heat treatment methods) in the FDM process, and crystallinity and mechanical properties. Vaezi et al. [16] investigated the mechanical properties of extrusion freeformed porous PEEK parts. Sun et al. [17] analyzed the effect of temperature on the mechanical properties and forming precision. Yang et al. [18] investigated the relationship among various thermal processing conditions in the FDM process, and mechanical properties. Gianluca et al. [19] investigated the tensile properties of the PEEK printed specimens for FDM printing technology. It was verified that PEEK has excellent mechanical properties compared with other filament materials. The mechanical properties of the 3D-printed parts are important indices for evaluating printing quality. However, it has not been sufficiently studied. Therefore, it is important to evaluate the mechanical properties of a new FDM technique for PEEK.

The main purpose of the present paper is to optimize the process parameters of FDM for PEEK components. A quadrature design of four factors and three levels was taken to study the processing parameters effect on the tensile strength and microstructure, as applying the printing temperature, printing speed, layer thickness and filling ratio. Furthermore, the bending test and the impact test were also implemented to verify the optimum process parameters.

## 2. Materials and Experiments

**Figure 1:**
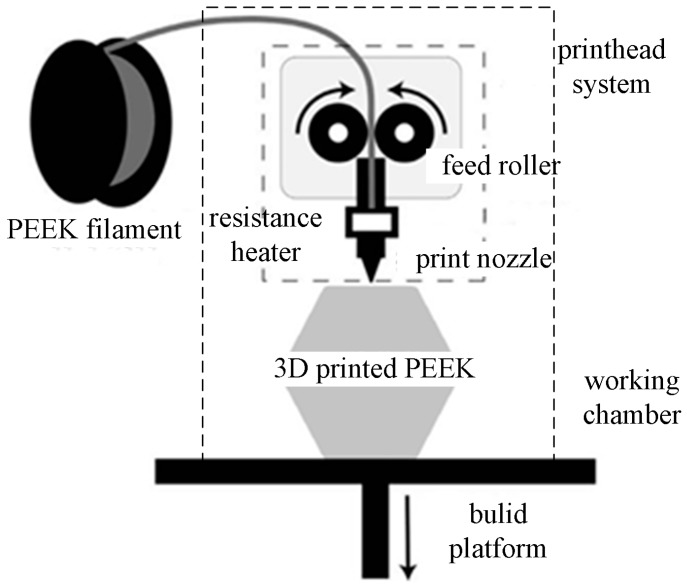
Schematic of customized polyether-ether-ketone (PEEK) fused deposition modeling (FDM) system.

It is generally accepted that higher or lower printing temperature, layer thickness or printing velocity may result in warpage of the specimen, which are directly related to properties of the FDM components [6]. The present research investigated the influence of the printing temperature, printing speed, layer thickness and filling ratio on the mechanical properties of the PEEK specimens. Specimens were made from 1.75 mm PEEK-1000 bar (Zhongshan Yousheng Plastic materials Co., Ltd., Zhongshan, Guangdong) and the custom-built FDM equipment, as shown in Figure 1.

Optimum FDM process parameters of PEEK tensile specimens were selected by the orthogonal test of four factors and three levels, with the printing temperature, printing speed, layer thickness and filling ratio, represented as A, B, C and D respectively. In the present study, the interactions among four parameters were neglected in order to simplify the test. Simplification has been proved actually feasible by the previous literature [10,19,20]. The orthogonal factor level table is shown in Table 1. L9 orthogonal array design was selected to improve experimental efficiency, as shown in Table 2. To enhance the experimental precision and the reliability of statistical analysis, as well as to eliminate the interference of experimental error, each test was conducted three times. Tensile properties of the PEEK specimens were measured with the universal material experiment machine (KL-WS-30S, Dongguan Kunlun Instrument Co., Ltd., Dongguan, Guangdong) by ISO 178:2001 Standard Test Method. The micro-structure and morphology of tensile specimens were examined using scanning electron microscopy (SEM) (TESCAN VEGA3, Brno, Czech Republic).

**Figure 2:**
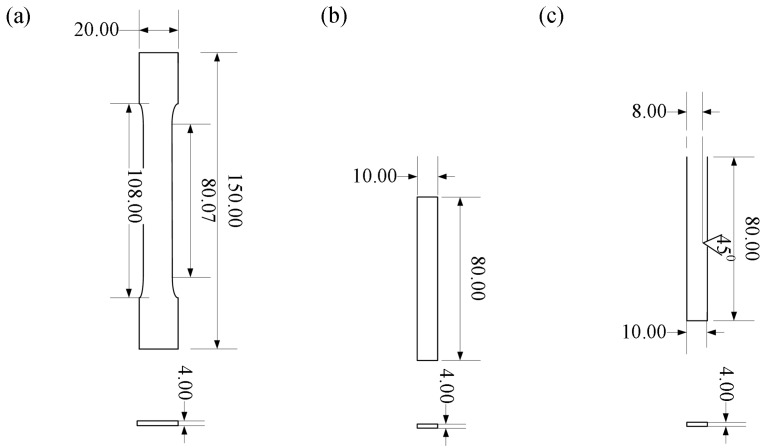
Geometric models of the mechanical test specimens. (**a**) Tensile specimen; (**b**) Bending specimen; (**c**) Impact specimen.

The impact test and bending test were conducted on the printed specimens by using the optimized processing parameters. The impact test was carried out with an impact velocity of 3.5 m/s, impacting angle of 150° and impact energy of 2.5 J. Impact testing specimens were designed according to ISO 179-1:2000. The three-point bending tests were performed according to the ISO 527-2:1993 with the loading rate of 2 mm/min. Both the impact test and three-point flexural test were also conducted using KL-WS-30S universal material experiment machine (KL-WS-30S, Dongguan Kunlun Instrument Co., Ltd., Dongguan, Guangdong). The geometric models are shown in Figure 2.

## 3. Results and Discussion

**Figure 3:**
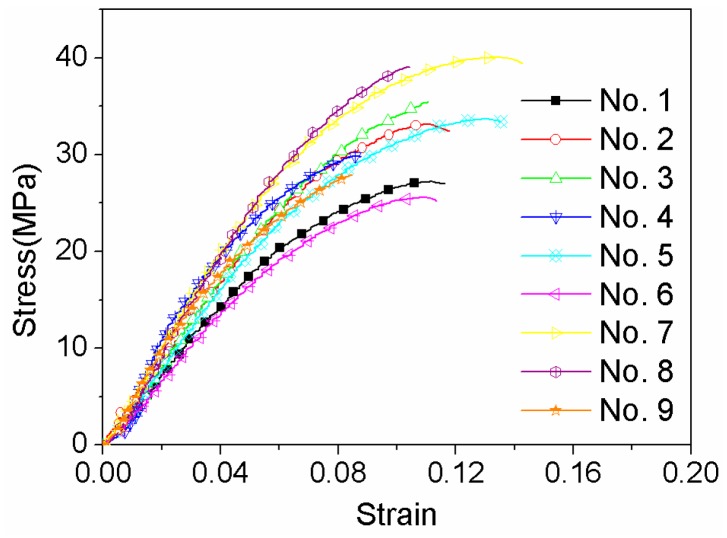
Stress-strain curves of PEEK FDM tensile specimens designed in Table 2.

The stress-strain relationship was calculated, as shown in Figure 3. The elastic modulus, tensile strength, elongation for different process parameters are listed in Table 3. The maximum tensile strength is 40.0 ± 4.4 MPa, which is lower than that reported for printed PEEK with an infill density of 100% [14]. Normally, the mechanical properties are proportional to the infill density during FDM process. The tensile strength dropped to 49.2 MPa when the infill density decreased to 80% in ref. [16], which would still maintain an appropriate strength and cause lower weight of PEEK components.

In order to obtain the optimized process parameters, range analysis was used with the elastic modulus, tensile strength and elongation. The range analysis of orthogonal experiments is listed in Table 4, Table 5 and Table 6, respectively. In these tables, K_iij_ (i = 1, 2, 3) is the sum of the experimental results in terms of level i, k_ij_ is the mean of K_ij_, *R* is range, which can be calculated by

(1) $$R\;=\;\max\;(k_{ij})-\min(k_{ij})$$

As shown in the Table 4, four level ranges of tensile strength are largely consistent, which have almost the same influence on the tensile strength. It can be seen that the optimal combination of tensile strength is A3B2C3D3, which is corresponding to the printing velocity of 60 mm/s, layer thickness of 0.25 mm, printing temperature of 370 °C and filling rate of 60%. In contrast, four ranges of elongation have a significant difference, as shown in Table 5. The sequence according to the effect of the elongation is as follows: printing temperature, filling rate, layer thickness and printing velocity. It can be concluded that the optimal combination of elongation is A1B2C3D2, which is corresponding to the printing velocity of 20 mm/s, layer thickness of 0.25 mm, printing temperature of 370 °C and filling rate of 40%.

Table 6 shows the range analysis of elastic modulus. The optimal combination of the elastic modulus is A3B1C2D3, which is corresponding to the printing velocity of 60 mm/s, layer thickness of 0.2 mm, printing temperature of 360 °C and filling rate of 60%. The sequence according to the magnitude of the elastic modulus’ range is as follows: filling rate, printing velocity, printing temperature and layer thickness.

It can be concluded that the optimal tensile properties vary with different process parameters. Therefore, the influence of these factors on the tensile properties should be comprehensively considered in determining the optimal printing parameters. Factor A exerted the greatest influence on the tensile strength. Moreover, it is of secondary importance to the elastic modulus. Therefore, the optimal choice of factor A is A3. In the same analysis, the optimum of factor B, C and D is B1, C3 and D2, respectively. From the above, the optimal combination of printing parameters is A3B1C3D2, which corresponds to the printing velocity 60 mm/s, layer thickness 0.2 mm, printing temperature 370 °C and filling rate 40%.

**Figure 4:**
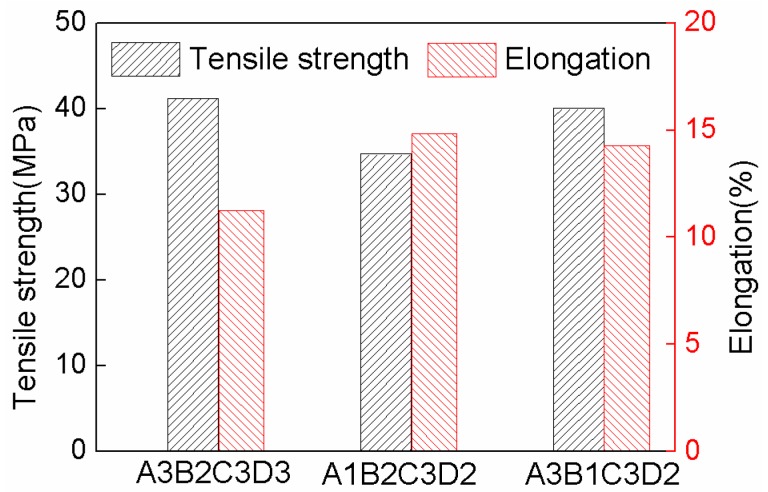
Comparison on tensile properties for different printing parameters.

The mechanical tests show that the tensile strength of No. 7 and 8 is higher, and elongation of No. 5 and No. 7 is greater. It can be concluded that the mechanical properties of No. 7 are the best in this investigation. It is consistent with the range analysis results of orthogonal test. In order to verify the results, the tensile specimens were prepared by using optimized parameters of A3B2C3D3 and A1B2C3D2. Figure 4 shows the comparison on tensile strength and elongation for different printing parameters. The tensile strength increased to 41.2 MPa using A3B2C3D3 processing parameters which is only 2.8% higher than that of No. 7 specimen, while, the elongation is lower by 21.1%. Likewise, when the A1B2C3D2 parameters was applied, the elongation could be improved to 14.8%, but the tensile strength is lower by 13.3% comparing to that of No. 7 specimen. Therefore, it can be concluded that the excellent integrated tensile properties were obtained using the No. 7 FDM processing parameters (A3B1C3D2).

The reason is that too high or too low process parameters may exert poor mechanical performance. Although, the rapid printing speed could improve the printing efficiency, the extrusion materials do not display enough plasticizing effect. If the printing speed is too slow, the fuse filament diameter will be uneven, and the fusion bonding would be much more difficult. The printing temperature also has a significant effect on the level of crystallinity, and thus the mechanical properties [17]. Too high extrusion temperature may cause the material degradation or extrudate, which cannot retain its shape upon deposition, resulting in filament deformation and dimensional inaccuracy. However, if the extrusion temperature is not high enough, the material does not have enough time to get fully melted which could result in the nozzle clogging. Layer thickness affects the width and speed of filament, and thus bonding and mechanical properties. The filling rate has a direct effect on the real section area of FDM components and the tensile stress.

**Figure 5:**
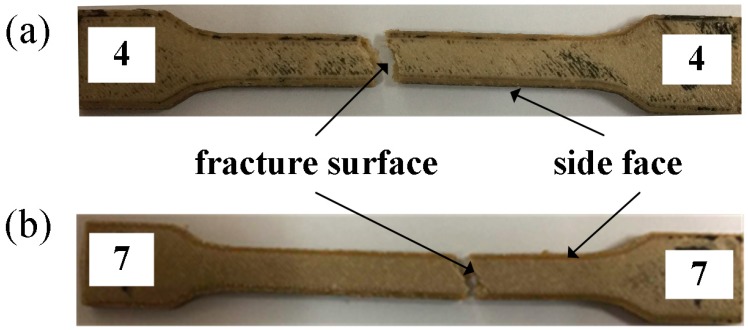
Fractured tensile specimens. (**a**) No. 4 specimen in Table 3; (**b**) No. 7 specimen in Table 3.

In this investigation, No. 4 and No. 7 specimens were selected to analyze the effect of process parameters on tensile properties. Figure 5 shows the fractured PEEK specimens after the tensile test. The elongation rate is 8.8% and 14.3% respectively. In addition, No. 7 specimen has higher tensile strength than No. 4 specimen.

**Figure 6:**
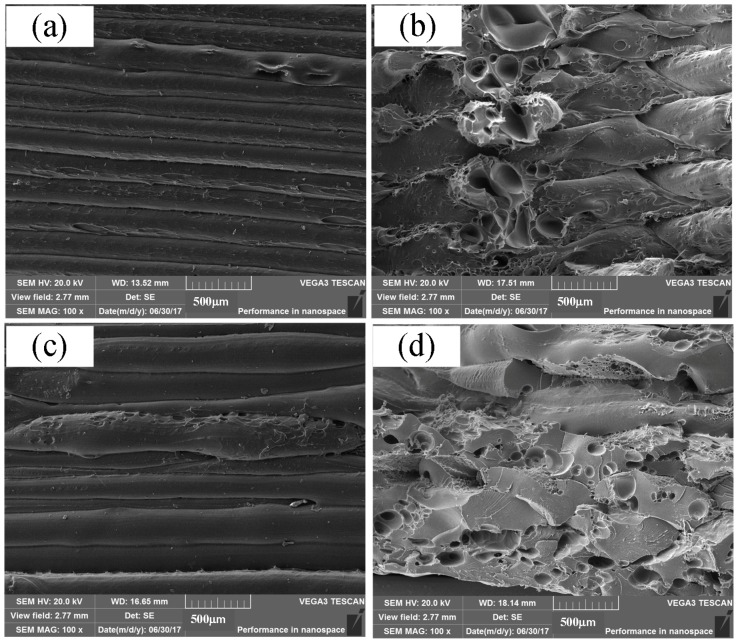
Microstructure and fracture surface. (**a**) side face microstructure of tensile specimen No. 7; (**b**) No. 7 fractured surface of tensile sample No. 7; (**c**) side face microstructure of tensile sample No. 4; (**d**) fractured surface of tensile sample No. 4.

Figure 6 shows the microstructures of two tensile PEEK specimens. It is evident that the lower strength specimen has more defects, such as air gaps and fusion line between infill filaments. It can be seen from Figure 6a that the interlamellar connectivity shows good fusion effects for No. 7 specimen, which is evidence of uniform fusion PEEK wire deposition during the FDM process. In FDM, the first deposited layer was between the aluminum build plate and fused PEEK wire, the connectivity basically depends on the subcooled temperature among the fused wire temperature, build plate temperature and working chamber temperature. In this investigation, the build plate temperature and working chamber temperature were set at 95 °C and 150 °C respectively and kept stable considering the high conductivity of aluminum alloy, which could develop a dense joining on the base plate. For the above layers, the printing coherence and connectivity primarily resulted from the fused temperature and deposited speed listed in Table 4. By comparing with the No. 4 specimen microstructure in Figure 6c, it can be concluded that the lower fused temperature and deposited speed could result in more slag inclusion, micro-pores and un-uniform deposition.

Figure 6b also shows that this region possesses excellent filament-to-filament and interlayer bonding. Furthermore, the fracture cross-sections show the failure mechanism. The center of the specimen is the initial failing zone for air gaps. This is contrast to the SLS on PEEK powders, which had a high porosity and many connected pores. In the SLS processing, the feedstock powders are always not totally molten or flatten in the as-sprayed coatings. The powders are only partially molten in the flame spraying process and incompact connected with each other when sprayed on the substrate or on the pre-deposited coating [21]. SLS process boundaries are shown concerning laser sintering temperature and energy input. The powder morphology and powder preparation also have great influence on the mechanical performance of the composite. In this investigation, the as-received PEEK wires were molten and extruded in the nozzle during the FDM process, which could result in a relatively tight connection between the layers by extrusion force. Meanwhile, PEEK powders were only melted by laser in the SLS, which tends to higher porosity among particles [4].

According to the analysis above, the optimal process parameters are set as printing speed of 60 mm/s, layer thickness of 0.2 mm, temperature of 370 °C and filling ratio of 40%. Both the three-point bending test and impact test were also performed to verify the mechanical properties.

**Figure 7:**
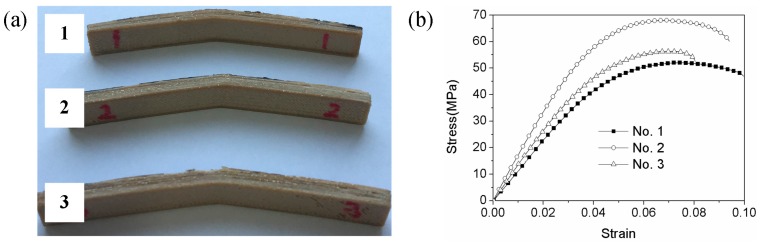
Bending test tests of PEEK FDM specimens. (**a**) Fractured bending specimens; (**b**) Bending stress-strain curves.

Figure 7a shows that the fractured bending specimens. The corresponding bending stress-strain curves are shown in Figure 7b. The maximum bending strength and bending modulus are 68.2 MPa and 1658.6 MPa, respectively. It can be concluded from Figure 6 that bending strength is greater than the tensile strength for the same process parameters. The reason for this is that the specimens are subjected to both tensile and compressive stresses during the bending tests. Moreover, the quality of the interlayer bonding has a great influence on flexural properties, which mainly correspond to the layer thickness [10]. It also can be seen from Figure 6 that the failure strength is 60.1 Mpa. The difference between the bending strength and the failure strength show the plastic deformation ability of the specimen which indicates the present specimen can sustain certain plastic deformation.

**Figure 8:**
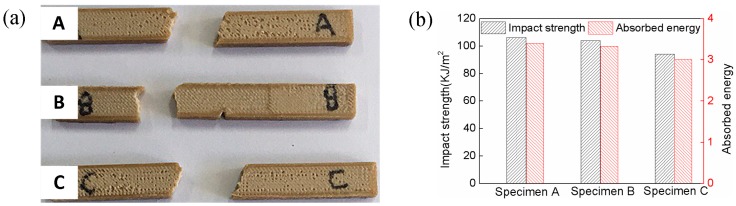
Impact test of PEEK FDM specimens. (**a**) Fractured impact specimens; (**b**) Impact strength and absorbed energy.

Figure 8 shows the impact test results. The mean impact strength and the absorbed energy of three specimens are 101.2 KJ/m^2^ and 3.25 J, respectively. In general, the impact strength of ABS approximates 15~40 KJ/m^2^. It is evident that the impact strength of PEEK is better than the common thermoplastic [22]. Moreover, the raster orientations have a significant effect on the absorbed energy, and the present specimens absorbed more energy comparing to the previous literature results for the same raster orientation [15]. Two main factors of fracture properties are the layer thickness and infilling rate. The high-filling and low layer thickness could strengthen the structure by providing additional bulk and crack resistance to the specimens [7]. Therefore, low layer thickness is necessary to improve the fracture properties under the low infilling density.

## 4. Conclusions

The effect of FDM processing parameters on the mechanical properties of high temperature PEEK materials was first systematically investigated in this study, including the tensile, bending and impact tests. The influences of printing temperature, filling rate, layer thickness and printing velocity on tensile properties were analyzed by orthogonal test of four factors and three levels. The range analysis indicated that the optimal combination of tensile strength is printing speed of 60 mm/s, layer thickness of 0.25 mm, printing temperature of 370 °C and filling rate of 60%, the optimal combination of elongation rate is set as printing speed of 20 mm/s, layer thickness of 0.25 mm, printing temperature of 370 °C and filling rate of 40%, and the optimal combination of elastic modulus is printing velocity of 60 mm/s, layer thickness of 0.2 mm, printing temperature of 360 °C and filling rate of 60%. The influence sequence on tensile properties was varied. According to the comprehensive analysis, the optimal combination of printing parameters is printing speed of 60 mm/s, layer thickness of 0.2 mm, temperature of 370 °C and filling ratio of 40%.

Furthermore, the tensile test indicated that the optimum tensile properties (tensile strength 40.0 Mpa, elongation 14.3%) could be obtained with the same parameters as the range analysis results. Good fusion effects and filament-to-filament and interlayer bonding at these parameters could be seen from SEM images. In addition, the printed bending and impact specimens with optimized parameters were performed. The flexural strength and impact strength are 68.2 Mpa and 101.2 KJ/m^2^, respectively, which is satisfactory for high performance engineering plastics. Further research is necessary to discuss the effects of other parameters, including the raster angle, orientation and diameter of the nozzle. Moreover, the interactions between different parameters should be considered in the future experimental design.

## Figures and Tables

**Table 1:** Orthogonal factor level table for PEEK FDM.

Level	Factor			
	Printing Speed (mm/s) A	Layer Thickness (mm) B	Printing Temperature (°C) C	Filling Ratio (%) D
1	20	0.20	350	20
2	40	0.25	360	40
3	60	0.30	370	60

**Table 2:** L9 orthogonal array design for PEEK FDM.

No.	Factor			
	A	B	C	D
1	20	0.20	350	20
2	20	0.25	360	40
3	20	0.30	370	60
4	40	0.20	360	60
5	40	0.25	370	20
6	40	0.30	350	40
7	60	0.20	370	40
8	60	0.25	350	60
9	60	0.30	360	20

**Table 3:** Tensile properties of orthogonal array designed specimens.

No.	Factor				Tensile Strength (MPa)	Elongation (%)	Young’s Modulus (MPa)
	A	B	C	D			
1	1	1	1	1	27.2 ± 1.5	11.6 ± 1.1	379.9 ± 20.0
2	1	2	2	2	33.2 ± 7.3	11.8 ± 1.4	416.3 ± 20.6
3	1	3	3	3	35.5 ± 5.0	11.1 ± 1.9	453.8 ± 91.3
4	2	1	2	3	29.9 ± 4.2	8.8 ± 1.3	576.5 ± 49.9
5	2	2	3	1	33.7 ± 3.2	13.5 ± 1.3	423.6 ± 15.2
6	2	3	1	2	25.6 ± 1.5	11.3 ± 0.7	355.2 ± 25.0
7	3	1	3	2	40.0 ± 4.4	14.3 ± 1.1	522.9 ± 32.1
8	3	2	1	3	39.1 ± 5.2	10.4 ± 0.9	533.1 ± 51.4
9	3	3	2	1	27.9 ± 1.5	8.5 ± 0.8	475.7 ± 43.2

**Table 4:** Range analysis of tensile strength.

Range	A	B	C	D
K_1j_	95.8	97.1	91.9	88.8
K_2j_	89.1	105.9	98.8	98.8
K_3j_	107.1	89.0	109.2	104.4
k_1j_	31.9	32.4	30.6	29.6
k_2j_	29.7	35.3	32.9	32.9
k_3j_	35.7	29.7	36.4	34.8
R	6.0	5.6	5.8	5.0
optimum levels	A3	B2	C3	D3
optimum assembly	A3B2C3D3			
order of priority	A C B D			

**Table 5:** Range analysis of elongation.

Range	A	B	C	D
K_1j_	34.5	34.6	33.4	33.6
K_2j_	33.6	35.8	29.0	37.4
K_3j_	33.2	30.9	38.9	30.3
k_1j_	11.5	11.5	11.1	11.2
k_2j_	11.2	11.9	9.7	12.5
k_3j_	11.1	10.3	13.0	10.1
R	0.4	1.6	3.3	2.4
optimum levels	A1	B2	C3	D2
optimum assembly	A1B2C3D2			
order of priority	D C B A			

**Table 6:** Range analysis of elastic modulus.

Range	A	B	C	D
K_1j_	1250.1	1479.3	1268.2	1279.2
K_2j_	1355.3	1373.0	1468.5	1294.4
K_3j_	1531.6	1284.7	1400.3	1563.5
k_1j_	416.7	493.1	422.7	426.4
k_2j_	451.8	457.7	489.5	431.5
k_3j_	510.5	428.2	466.8	521.2
R	93.9	64.9	66.8	94.8
optimum levels	A3	B1	C2	D3
optimum assembly	A3B1C2D3			
order of priority	D A C B			
